# Mitochondrial microRNAs (mitomiRs) as emerging biomarkers and therapeutic targets for chronic human diseases

**DOI:** 10.3389/fgene.2025.1555563

**Published:** 2025-04-25

**Authors:** Andrea Méndez-García, Marely Abigail García-Mendoza, Carmila Patricia Zárate-Peralta, Fernanda Valeria Flores-Perez, Luis Fernando Carmona-Ramirez, Surajit Pathak, Antara Banerjee, Asim K. Duttaroy, Sujay Paul

**Affiliations:** ^1^ Tecnologico de Monterrey, School of Engineering and Sciences, Queretaro, Mexico; ^2^ Tecnologico de Monterrey, School of Engineering and Sciences, Ciudad de Mexico, Mexico; ^3^ Tecnologico de Monterrey, School of Engineering and Sciences, Nuevo Leon, Mexico; ^4^ Chettinad Academy of Research and Education (CARE), Chettinad Hospital and Research Institute (CHRI), Department of Medical Biotechnology, Faculty of Allied Health Sciences, Chennai, India; ^5^ Department of Nutrition, Institute of Basic Medical Sciences, Faculty of Medicine, University of Oslo, Oslo, Norway

**Keywords:** mitochondria, micrornas, mitomirs, chronic diseases, gene regulation, biomarkers, therapeutics

## Abstract

Mitochondria are membrane-bound cell organelles that undertake the majority of the energetic and metabolic processes within the cell. They are also responsible for mediating multiple apoptotic pathways, balancing redox charges, and scavenging reactive oxygen species. MicroRNAs, which are short, non-coding RNAs widely known for regulating gene expression at the post-transcriptional level, regulate many of these processes. The specific microRNAs that directly or indirectly control mitochondrial dynamics are called mitochondrial miRNAs (mitomiRs). The broadest classification of this type of ncRNA encompasses nuclear-encoded miRNAs that interact with cytoplasmatic mRNAs associated with mitochondrial activity. At the same time, a more specific subset comprises nuclear-encoded miRNAs that translocate into the mitochondria to interact with mRNAs inside of this organelle. Finally, the smallest group of mitomiRs includes those codified by mtDNA and can regulate endogenous mitochondrial transcripts or be transported into the cytoplasm to modulate circulating mRNAs. Regardless of the origin or action mechanism, mitomiRs have been recently recognized to have a key role in the progression of a variety of chronic disorders, such as neurodegenerative and cardiovascular diseases, diabetes, asthma, depression, and even cancer. All of these progressive pathologies have been tightly linked to mitochondrial dysregulation. They are further associated with an aberrant expression of specific miRNAs that regulate cellular metabolism, positioning mitomiRs as reliable biomarkers for diagnosing several chronic diseases. These molecular indicators have also provided insights into how these conditions progress, allowing for the development of different miRNA-based treatment strategies that target dysregulated mitochondrial-related genes, reestablishing their baseline activity and restricting further disease progression.

## 1 Introduction

The mitochondrion is an organelle that holds primary significance in almost 90% of cellular energetic processes (Grasso et al., 2020; Feng et al., 2022). It is best known for being responsible for the aerobic production of adenosine triphosphate (ATP) through oxidative phosphorylation (OXPHOS) and Krebs cycle processes (Grasso et al., 2020; Gasmi et al., 2021). At a broad level, the outer phospholipid membrane delimits mitochondria from the cytoplasm. Inside, the matrix is surrounded by the inner phospholipid membrane, where OXPHOS occurs. Both membranes are separated by the intermembrane space (Glancy et al., 2020; Rigoulet et al., 2020; Harrington et al., 2023).

It is strongly hypothesized that this organelle evolved from the integration of an alphaproteobacterium into an anaerobic host archaeon, where the bacterium aerobically produced energy for the archaeon, giving place to an interdependent relationship (Purohit and Saini, 2021; Vringer and Tait, 2022; Murphy and O’Neill, 2024). This endosymbiotic background would hold the explanation for why mitochondria possess their DNA, which is, in fact, very similar to bacterial DNA. The modern structure of the evolved bacterium, now referred to as mitochondrion, contains the mitochondrial DNA (mtDNA) within its matrix, where 2–10 copies of the 16,569 base-pair genome are found. This circular double-stranded DNA is replicated by DNA polymerase γ and has been found to encode 37 genes: 22 tRNAs, 13 proteins involved in electron transfer, and 2 rRNAs (Purohit and Saini, 2021; Gowda et al., 2022; Harrington et al., 2023). Unlike the nuclear genome, mtDNA is unidirectionally inherited from the maternal side (Munasinghe and Ågren, 2023). It is believed that this dynamic allows the degradation of the genetic material from the male gamete as the embryo develops to impede the transmission of fatal mutations from the paternal mtDNA (Melo and Camargo, 2021).

Mitochondria contribute significantly to maintaining redox homeostasis, mediating apoptosis pathways, controlling cell proliferation, transmitting signals (Liao et al., 2022), regulating calcium, scavenging and producing free radicals, and differentiating cells (Casanova et al., 2023). Given the complexity and variety of mitochondrial activities, mtDNA extensively, but not exclusively, controls these processes, as many of them are also mediated by the nuclear genome, building sophisticated crosstalk between both genetic materials that effectively coordinate to carry on all mitochondrial functions (Scarpulla, 2011; Jusic and Devaux, 2020). The dysregulation of genes associated with energetic and metabolic cellular processes disrupts the mitochondria’s optimal performance, which potentially triggers cardiovascular diseases, type II diabetes mellitus (T2DM), cancer, neurodegeneration, and other chronic pathologies (Purohit and Saini, 2021; Gohel and Singh, 2022).

Although multiple mechanisms that cause mitochondrial dysregulation have been explored, emerging evidence suggests that microRNAs (miRNAs) significantly influence several mitochondrial processes (Purohit and Saini, 2021; Ren et al., 2023). For instance, miR-181c binds to mitochondrial genomic transcript cytochrome c oxidase subunit 1 (*mt-COX1*) after it translocates into the mitochondria, triggering the upsurge of ROS and, at the same time, affecting the function of Specificity Protein 1 (Sp1) and mitochondrial calcium uptake 1 protein (MICU1), which ultimately contributes to cardiac dysfunction (Roman et al., 2020). On the other hand, miR-2392 is able to inhibit the activity of mitochondrial complexes I, III, and IV by negatively regulating the transcription of mitochondrial DNA, leading to a metabolic shift toward glycolysis associated with chemoresistance in tongue cancer (Fan et al., 2019).

MiRNAs are a type of small (18–25 nt) non-coding RNA (ncRNA) that operate as gene regulators at the post-transcriptional level (Benavides-Aguilar et al., 2023; Bravo-Vázquez et al., 2023a; Osorio-Pérez et al., 2023; Castañón-Cortés et al., 2024). These endogenous and highly conserved single-stranded RNA molecules bind to the 3′ UTR complementary region of messenger RNA (mRNA) sequences with the purpose of inhibiting their expression either by degrading them or physically blocking the transcription process (Naeli et al., 2023). Interestingly, more recent evidence has suggested that miRNAs can bind to the 5′UTR region, the coding section, and even to the promoter sequences (Gohel and Singh, 2022). Even though the vast majority of miRNAs act upon cytoplasmic mRNAs, emerging studies have confirmed the presence of these master regulators inside the mitochondria (Giordani et al., 2021) and, most importantly, their capacity to regulate the expression of not only the mtDNAs-transcribed mRNAs but also the cytoplasmatic mRNAs. Stated otherwise, miRNAs encoded by the nuclear genome are able to enter mitochondria and regulate the mRNAs circulating within this organelle, regardless of whether these messengers were initially transcribed from the nuclear or mitochondrial genetic material (Pozzi and Dowling, 2022). This group of microRNAs is denominated mitochondrial microRNAs (mitomiRs). Additionally, several studies have provided evidence of miRNAs encoded by the mtDNA; however, they represent the smallest percentage within the mitomiRs classification. The most extensive two categories of mitomiRs correspond to the microRNAs of nuclear origin that are transported into the mitochondrial matrix to regulate the expression of mRNAs present in this compartment and to the extensive pool of nuclear-encoded miRNAs that target cytosolic mRNAs accountable for regulating mitochondrial functions directly or indirectly (Duroux-Richard et al., 2021; Purohit and Saini, 2021; Gohel and Singh, 2022; Kuthethur et al., 2022; Patel et al., 2023).

**FIGURE 1:**
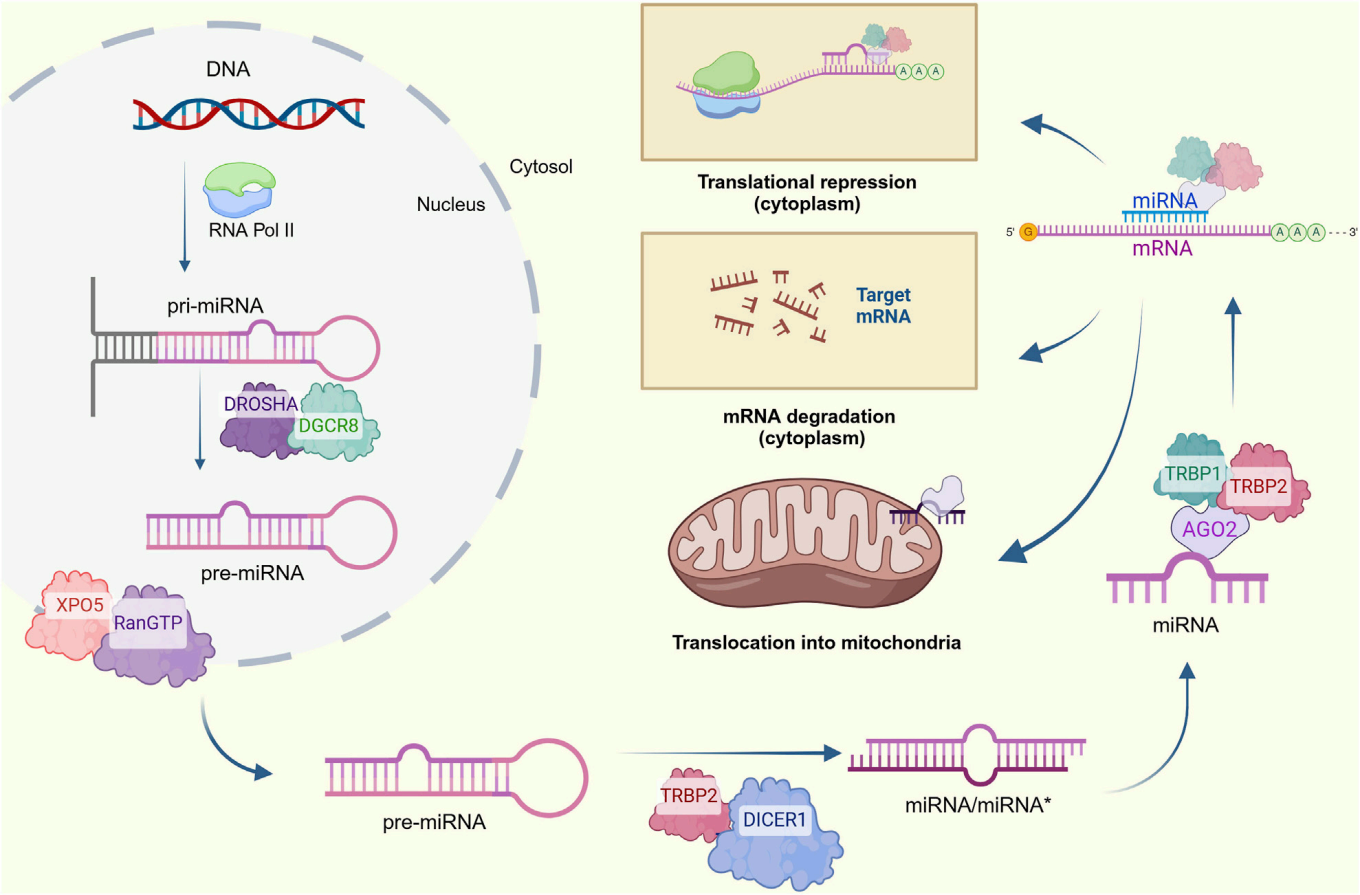
Canonical process for miRNA biogenesis. Pri-miRNAs are transcribed from nuclear DNA and cleaved into pre-miRNAs, which are then exported to the cytosol and processed to form a miRNA/miRNA* duplex. The complementary strand is degraded for the mature miRNA to form the RISC complex and target mRNAs to decrease their expression *via* translational repression of degradation of cytoplasmatic transcripts. An alternative translocation to mitochondria is also considered (Created at https://BioRender.com).

The canonical biogenesis of nuclear-encoded miRNAs (Figure 1) begins with the transcription of a miRNA-codifying gene into a primary miRNA (pri-miRNA) by RNA polymerase II (Ruiz-Manriquez et al., 2022; Ledesma-Pacheco et al., 2023). The resulting stem-looped structure (500–3,000 nucleotides-long) containing a 3′ polyadenylated tail and a 5′ cap is then cleaved at the stem by RNAse III DROSHA and its cofactor RNA binding protein DiGeorge syndrome critical region 8 (DGCR8), also referred to as Pasha, to release a 60–70-nucleotide miRNA precursor (pre-miRNA) (Catanesi et al., 2020; Barrey et al., 2011). This hairpin structure further interacts with exportin-5 (XPO5) and the Ras-related nuclear protein guanosine triphosphate (RanGTP) to be transferred from the nucleus to the cytoplasm. Then, the pre-miRNA is processed by RNAse III DICER1 and its cofactor transactivation-responsive RNA-binding protein (TRBP), generating a 20-22-nucleotide miRNA duplex constituted by a mature miRNA guide strand and a complementary passenger strand termed as miRNA*; the latter is degraded, while the remaining mature miRNA binds with the Argonaute protein (AGO2), TRBP1 and TRBP2 to form the miRNA-induced silencing complex (miRISC) (Srinivasan and Das, 2015; Catanesi et al., 2020; Lao and Huyen Le, 2020).

At this point, two alternative pathways emerge: the regulation of cytoplasmic mRNA involved in mitochondrial functions or the translocation of miRNA into the mitochondria. For the first case, the 5′ end of the miRNA usually pairs with the 3′-UTR of the mRNA target or targets. A perfect affinity results in mRNA degradation, while imperfect pairing leads to translational inhibition (Lao and Huyen Le, 2020). In contrast, the specific mechanism by which the miRNAs translocate into the mitochondria remains undeciphered and seems to differ between species (Kim et al., 2017). Accumulating evidence has suggested that AGO2 has a key role in transporting miRNAs into mitochondrial compartments. In fact, the AGO2/miRNA complex-dependent mechanism is the most supported given the mitochondria’s targeting sequence located in the N-terminal of AGO2 and its enriched presence identified in the mitochondrial fraction of several molecular purifications (Bandiera et al., 2013; Srinivasan and Das, 2015; Shepherd et al., 2017). The entry of AGO2 together with the miRNA into the mitochondria has been explained by several potential mechanisms. The first one proposes the interaction of this complex with the translocase of the outer membrane (TOM20), a surface mitochondrial receptor, or with the Sorting and Assembly Machinery (SAM50) channel (Giordani et al., 2021; Jusic and Devaux, 2020). Another mechanism suggests the formation of processing bodies (p-bodies) by phosphorylation reactions, which might facilitate miRNA importation into the mitochondria (Bandiera et al., 2013; Macgregor-Das and Das, 2018; Giordani et al., 2021). Other complementary mechanisms indicate that the interaction between polynucleotide phosphorylases (PNPASEs) and RNA stem-loop secondary structures allows the transportation of pre-miRNAs into this organelle and, more interestingly, the later translocation of mature miRNAs into the mitochondrial matrix through the translocase of the inner membrane (TIM), again supported by AGO2 (Bandiera et al., 2013; Macgregor-Das and Das, 2018; Giordani et al., 2021; Gohel and Singh, 2022). Finally, highly conserved protein channels around the mitochondrial membrane named porins are also considered to control miRNA trafficking (Bandiera et al., 2013; Giordani et al., 2021).

**FIGURE 2:**
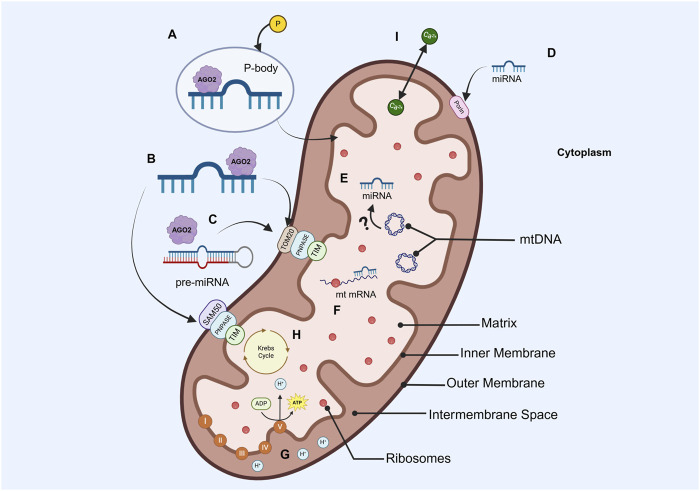
Simplified diagram of the mitochondrion structure, miRNA importation and silencing mechanisms, and most popular energetic processes **(A)** Nuclear-encoded miRNAs in a p-body, which would facilitate the entrance of miRNAs into the mitochondria **(B)** AGO2/miRNA complex interaction with TOM20 or SAN50 pores in the outer membrane, PNPASE in the intermembrane space, and TIM in the inner membrane for their importation to the matrix **(C)** AGO2/Pre-miRNA association with PNPASEs allows the translocation of precursors into the mitochondria **(D)** Nuclear-encoded miRNAs importation to the mitochondria *via* porins **(E)** mtDNA-encoded miRNAs, whose biogenesis process is not yet elucidated **(F)** Transcripts encoded by mtDNA can be silenced by imported or endogenous miRNAs **(G)** OXPHOS **(H)** Krebs cycle **(I)** Calcium transportation (Created in https://BioRender.com).

Once the mitomiRs have been successfully imported, they regulate mtDNA expression in various manners. The first one describes the direct binding of the mitomiRs to the mtDNA, while another one suggests immature mitochondrial transcript repression by direct mRNA-miRNA binding. Ultimately, mitomiRs could silence mature mitochondrial mRNA by impeding the correct mitochondrial ribosome function (Gohel and Singh, 2022). The biogenesis, translocation, and gene-regulation mechanisms of nuclear- and mtDNA-encoded mitomiRs are depicted in Figure 2.

Although there are recent research findings of miRNAs encoded by mtDNA such as has-miR-4461, has-miR-4463 (Ren et al., 2023), has-miR-4484, and has-miR-4485 (Kuthethur et al., 2022; Tokgün and Tomatir, 2023), there is yet no evidence of their concrete biogenesis pathway (Santovito and Weber, 2022; Ren et al., 2023; Roiz-Valle et al., 2023). Nonetheless, the previously mentioned mitochondrial-encoded miRNAs have shown to be homologous with the mtDNA regions NADH dehydrogenase subunits 4L–5L (*ND4L-5L*), crucial in the electron transport chain (Destiarani et al., 2020), L-open reading frame (*L-ORF*), and *16S rRNA*, which plays a significant role in the synthesis of mitochondrial ribosomes, respectively (Tokgün and Tomatir, 2023).

The relationship between mitochondrial dysfunction and the development of cardiovascular, neurodegenerative, inflammatory, and other chronic diseases such as cancer and T2DM has been extensively demonstrated (Diaz-Vegas et al., 2020). Still, it was only recently established that miRNAs are capable of controlling the metabolic functions of the mitochondria and hence have significant impacts on these health conditions (Destiarani et al., 2020; Grasso et al., 2020; Ortega-Trejo et al., 2022; Santovito and Weber, 2022; Vringer and Tait, 2022; Harrington et al., 2023).

Additionally, growing evidence indicates that lifestyle choices and environmental factors can also influence mitomiR expression and therefore, have multiple effects in the development of a variety of chronic diseases. For instance, several preclinical and clinical studies of patients with sarcopenia have demonstrated that maintaining physical exercise improves mitochondrial functions by modifying the expression of twelve miRNAs. In detail, clinical studies evidenced that miR-31 was drastically decreased when performing endurance exercise, improving mitochondrial biogenesis and function. Additionally, during resistance exercise, downregulation of miR-1, -15a, −23a, −126, −130a, and −503 improves muscle protein synthesis mitochondrial density and enhances ATP production (Long et al., 2023). Within the nutrigenomics area, there has been a solid relationship among diet, obesity, miRNA expression, and mitochondrial functioning. In fact, a high-fat diet in mice with miR-181a overexpression was shown to reduce body weight, while its knockdown exhibited increased body weight, strongly suggesting the effect this miRNA has in adipogenesis through mitophagy. More compellingly, the presence of miR-21 in adipose tissue has proved to promote oxidative stress and inflammation. In diet-induced obesity, this miRNA is especially elevated inside white adipose tissue (Murri and Azzouzi, 2018).

The robust and well-established associations between mitochondrial-associated chronic diseases and differential mitomiR expression underscore the potential of these ncRNAs as reliable biomarkers for the diagnosis of these pathologies, as well as promising targets for the development of novel therapeutic strategies. When compared to other existing biomarkers and therapeutic approaches, mitomiRs specifically offer additional advantages as they are more specifically directed to the metabolic perspective of the disease, which covers a broad role in both its onset and progression, especially because they offer comprehensive insights into the disruptions in carbohydrate and lipid metabolism. In cancer, for example, they regulate multiple oncogenic signaling pathways and directly target essential proteins involved in tumor metabolism (Ortega et al., 2020). A deep and precise understanding of the particular mitomiRs’ regulatory roles will definitively pave the way for the emergence of state-of-the-art prognostic and therapeutic approaches for chronic diseases, contributing to an improvement in patients’ quality of life.

## 2 MitomiRs as biomarkers in common human chronic diseases

MicroRNAs have acted as reliable biomarkers for diseases, as their usual expression becomes modified when the organism is under stressful conditions (Wu et al., 2021). Cardiovascular and neurodegenerative diseases, as well as T2DM and cancer, are pathologies that have been closely associated with mitochondrial dysfunction (Bienertova-Vasku et al., 2013; Gowda et al., 2022; Lin et al., 2022; Ao et al., 2023). More importantly, there is evidence of differentially expressed miRNAs involved in regulating the mitochondrial metabolism in these health conditions, whether they are localized inside the mitochondria (direct regulators) or in the cytoplasm (indirect regulators) (Kuthethur et al., 2022). In the present review, we compiled the most recent findings of mitomiRs whose expression was found dysregulated in the most common neurodegenerative and cardiovascular diseases, as well as in T2DM, cancer, depression, and asthma, which makes them prospective biomarkers and therapeutic targets for each condition.

### 2.1 MitomiRs in cardiovascular diseases

Cardiovascular chronic diseases comprise atherosclerosis, hypertension, heart failure, coronary and peripheral artery disease, cardiomyopathy, and congenital heart defects, among others (Nisselrooij et al., 2020; Shao et al., 2020; Brouwers et al., 2021; Hollenberg and Singer, 2021; Libby, 2021; Lopaschuk et al., 2021). They are related to the building of lipid deposits in the arteries, as in the case of atherosclerosis, coronary, and peripheral artery disease (Libby, 2021; Shao et al., 2020), or the incapacity of the heart to pump blood regularly, as it happens for hypertension, heart failure, cardiomyopathy and heart defects (Nisselrooij et al., 2020; Brouwers et al., 2021; Hollenberg and Singer, 2021; Lopaschuk et al., 2021). For heart failure alone, its annual global prevalence is around 26 million people (Beezer et al., 2022), and it is expected to continue increasing in the following years. Meanwhile, 17.8 million deaths were registered in 2019 caused by cardiovascular diseases overall (Ao et al., 2023). More importantly, almost all cardiovascular chronic diseases are highly associated with obesity (Powell-Wiley et al., 2021), a condition present in nearly 42% of the worldwide population (Wong et al., 2020).

Accumulating evidence has shown that impaired functioning of the mitochondria is a starting point for the pathogenesis of cardiovascular diseases as it compromises optimal cellular respiration energy production and stimulates ROS generation, explicitly affecting endothelial cells in the vascular walls, triggering a series of biochemical and immune reactions that end up in the accumulation of lipids, release of apoptotic factors, oxidative stress, and insufficient energy production (Poznyak et al., 2020). Although the detailed mechanism on which mitomiRs contribute to developing cardiovascular diseases is still underexplored, it is mainly associated with an induction of ROS levels above the critical threshold, predominantly affecting the cardiomyocytes (Poznyak et al., 2020; Ao et al., 2023), making mitomiRs suitable biomarkers that would foreshadow chronic affections of the cardiovascular system.

In this context, Ding et al. identified miR-214 as a negative regulator of sirtuin 3 (*SIRT3*), which codifies for a mitochondrial protein deacetylase crucial for the regulation of fatty acid and glucose metabolism, redox homeostasis, and mitochondrial integrity. Reduced levels of SIRT3 in human hearts are linked to hyperacetylation of mitochondrial proteins and disrupted energetic metabolism, leading to structural and functional alterations in the heart, ultimately contributing to cardiac hypertrophy and heart failure. Illumina deep sequencing showed significant overexpression of miR-214 in the left ventricular tissue of cardiac hypertrophic mice models accompanied by a reduced expression of SIRT3; consequently, miR-214 inhibition using antagomiR-214 restored SIRT3 mitochondrial levels, reduced oxidative stress, promoted mitochondrial biogenesis, and improved heart function. This positioned miR-214 as a potential biomarker and a promising therapeutic target for chronic cardiovascular diseases linked to mitochondrial dysfunction and energetic metabolism disbalances (Ding et al., 2020).

A similar study utilized a murine model of cardiac hypertrophy induced by transverse aortic constriction (TAC) to explore the role of miR-27b-3p in cardiac remodeling, a condition that affects heart size, mass, and function. After evaluating conditions like myocardial fibrosis, hypertrophy, and cardiac inflammation in miR-27b-3p-knockout mice, the results of RNA transcriptome sequencing analyses and histological assessments revealed that the absence of miR-27b-3p exhibited a significant reduction in these pathological factors compared to the TAC group. In addition, enzyme-linked immunosorbent assay (ELISA) revealed that fibroblast growth factor 1 (*FGF1*) was a target gene of miR-27b-3p. The overexpression of this gene was associated with improved mitochondrial function and reduced hypertrophy, indicating that this factor could counteract the adverse effects seen in pathological cardiac hypertrophy and cardiac remodeling. The potential of miR-27b-3p as a biomarker for the diagnosis and monitoring of these conditions is highlighted, with significant clinical implications for the management of heart diseases (Li et al., 2022).

Pathological hypertrophy leads to cardiac dilation and heart failure. This process compromises mitochondrial structural integrity, disrupts cellular homeostasis, impairs mitochondrial calcium handling, and exacerbates oxidative stress. To explore the role of miRNA-7 in mediating cardiac hypertrophic response, Gupta et al. (2021) overexpressed this miRNA in the cardiomyocytes of C57Bl/6 mice. Their results of quantitative real-time PCRs and western blottings showed that miR-7 negatively regulated the erythroblastic oncogene B (*ERBB2*), an epidermal growth factor receptor associated with accelerated heart failure. Depletion of *ERBB2* led to alterations in the oxidative phosphorylation cycle, reduction of integral protein of mitochondrial membrane Growth hormone-inducible transmembrane protein (GHITM) amount, and cristae-less morphology, all of which corresponded to the usual phenotype of end-stage heart failure. Overall, overexpression of miR-7 suggested that this miRNA could be a useful biomarker for the identification of cardiac dilation and further heart failure (Gupta et al., 2021).

Yan et al. (2019) examined the role of miR-762 in mitochondrial dysfunction and ATP production during ischemic injury in myocardial cells, as it has been closely associated with the pathogenesis of cardiovascular disease. Its expression profile was analyzed in neonatal mouse cardiomyocytes subjected to anoxia/reoxygenation (A/R) as a model for ischemia. A significant upregulation of miR-762 was noticed within the mitochondria of this group. On the contrary, miR-762 knockdown effectively attenuated ischemia-induced damage by reducing elevated reactive oxygen species (ROS) production, attenuating decreased intracellular ATP levels, restoring mitochondrial complex I enzyme activity, and reducing apoptotic cell death. Furthermore, suppressing miR-762 *in vivo* reduced myocardial ischemia/reperfusion (I/R) injury, highlighting its pathological role. MiR-762 was also found to directly bind to the coding sequence of NADH dehydrogenase subunit 2 (*ND2*), and loss of expression of this protein mirrored the mitochondrial dysfunction observed in ischemia, including diminished ATP production, elevated ROS levels, and increased apoptosis. This study highlighted miR-762 as a key mediator of mitochondrial dysfunction in ischemia and a promising biomarker that might further result in therapeutic intervention.

Obesity induces significant mitochondrial dysfunction in cardiomyocytes, characterized by increased ROS production and elevated calcium levels in the mitochondrial matrix ([Ca^2+^]_m_), both of which contribute to heart failure progression. A study performed in 2020 explored the role of miR-181c, a miRNA encoded by the nuclear genome and translocated into the mitochondrial compartment of cardiomyocytes. The research employed miR-181c/d−/− mice in a high-fat diet to overexpress miR-181c via artificial ncRNA introduction. Results indicated that miR-181c directly bound to the *mt-COX1*, thereby reducing COX1 expression and consequently enhancing ROS production, as this gene has a key role in mitochondrial oxidative phosphorylation. Subsequently, ROS oxidized cysteine residues in the Sp1, a transcription factor that regulates the mitochondrial calcium uptake 1 protein MICU1 expression, which predominantly works in the heart. The interaction blockage between Sp1 and MICU1 contributed to an increase in the concentration of [Ca^2+^]_m_, establishing a conductive setting for cardiac dysfunction. The data presented compelling evidence that miR-181c is a reliable biomarker that indicates abnormal heart performance in obese patients, also offering a potential therapeutic target against heart disorders caused by being overweight (Roman et al., 2020).

### 2.2 MitomiRs in neurodegenerative diseases

Neurodegenerative diseases are pathologies that degrade neurons progressively. Some of these disorders are Parkingson’s, Alzheimer’s, and Huntington’s diseases, all of which share a close connection with elevated oxidative stress (Wang et al., 2020). Alzheimer’s disease (AD) is characterized by the loss of synaptic connections and the accumulation of amyloid β and phosphorylated τ, while Parkinson’s primary symptoms include tremors and muscular rigidity. Huntington’s consists of loss of coordination, involuntary movements, and other motor impairments (John et al., 2020). These conditions affect approximately 15% of the global population, which is expected to duplicate in the following decades due to the growing aging population (Schependom and Van & D’haeseleer, 2023).

MitomiRs are implicated in neuronal death caused by a dysregulation of the ROS-dependent apoptotic signaling pathways and in mitochondrial biogenesis. This process replenishes properly functioning mitochondria (Purohit and Saini, 2021). There are well-established purifying methods that allow the extraction of miRNAs present in the neuron synaptosome mitochondria and thus confirm the presence of differentially expressed mitomiRs that are further related to degenerative processes in the central and peripheral nervous systems (Gowda et al., 2022).

Parkinson’s disease (PD) is the second most common neurodegenerative disease in humans. In this regard, Baghi et al. (2020) investigated the association between miR-376a and key regulators involved in PD progression. Specifically, they focused on evaluating the expression of target genes peroxisome proliferator-activated receptor gamma coactivator 1-alpha (*PGC1α*), mitochondrial transcription factor A (*TFAM*), and glycogen synthase kinase 3 beta (*GSK3β*) in SHSY5Y cell line treated with 1-methyl-4-phenylpyridinium. RT-qPCR measured the expression levels of miR-376a and related genes, and the authors found that the downregulation of miR-376a is inversely correlated with the overexpression of *PGC1α* and *TFAM*. An elevated expression of the first one demonstrated to have neuroprotective effects against neurotoxins, while *TFAM* overexpression enhanced mitochondrial gene expression and boosted respiratory chain protein levels. This study suggested the role of miR-376a in PD pathogenesis through the regulation of mitochondrial function-related genes, proposing it as a potential biomarker for PD diagnosis (Baghi et al., 2020).

Later, that same research group highlighted miR-193b as a key regulator of the *PGC1α/FNDC5/BDNF* pathway, essential for mitochondrial function and neuroprotection. They found that miR-193b was overexpressed in peripheral blood mononuclear cells of PD patients, leading to sustained downregulation of *PGC1α*, Fibronectin type III domain-containing protein 5 (*FNDC5*), brain-derived neurotrophic factor (*BDNF*), and *TFAM*, exacerbating mitochondrial dysfunction and neuronal degeneration. Additionally, elevated ROS confirmed oxidative stress as a major driver of PD pathology. Here, findings suggest miR-193b as a potential biomarker for PD diagnosis, supported by significant inverse correlations between miR-193b and the aforementioned mitochondrial-related genes. Overall, the study emphasized the critical role of miR-193b in regulating mitochondrial health and its potential as a therapeutic target against PD (Baghi et al., 2021).

MirR-181 is a family of miRNA known for its ability to be translocated into mitochondria, where it plays a crucial role in regulating mitochondrial morphology and function. For instance, miR-181a is specifically involved in controlling mitochondrial activity linked to cellular senescence and aging, highlighting its importance in maintaining mitochondrial health and cellular homeostasis (Braicu et al., 2019). This miRNA is abundant in the brain, which plays a crucial role in respiration, biogenesis, and growth of neurites, which are nerve cell extensions. Interestingly, its dysregulation is linked with neurodegenerative diseases like Parkinson’s disease (PD). Stein et al. (2022) investigated the involvement of miR-181 in the degeneration of dopaminergic neurons (DA), which play a key role in motor control and other neurological processes that usually deteriorate during PD. In their study, adeno-associated viral vectors were employed as vehicles of miR-181a1/b2 for systemically introducing them into C57Bl/6J rodents. The results revealed that overexpression of mirR-181 was associated with a significant downregulation of *Gabra1*, involved in dopamine-driven movement; *Kcnj6*, which regulates neuronal excitability; and *Chchd10*, crucial for mitochondrial function, suggesting a link to neurodegeneration. The reduction of these genes negatively affected DA’s function and excitability, contributing to neurodegeneration and the appearance of PD motor symptoms. Furthermore, the inhibition of miR-181 appeared to offer a protective effect against neurotoxicity, potentially preserving the function and integrity of DA. However, further research is still required to understand how miR-181 influences neurodegeneration fully and its potential as a therapeutic target in treating PD (Stein et al., 2022).

The accumulation of dysfunctional mitochondria and ROS caused by unpaired mitophagy contributes to cellular damage and neurodegeneration. This clinical course has been associated with miR-218, as it has been stated that this miRNA regulates Parkin RBR E3 ubiquitin ligase (*PRKN*) by decreasing its mRNA and protein levels, affecting ubiquitylation signaling, crucial for triggering mitophagy. This gene has demonstrated key roles in PD, AD, Amyotrophic Lateral Sclerosis, and Huntington’s disease and in protecting neurons against neurotoxins and metallic ions. Rita et al. (2020) revealed that miR-218 targets cytosolic *PRKN* mRNA, blocking its translation and further translocating PRKN protein into the mitochondria. Furthermore, HEK293 transfected with miR-218 showed no reduction of TOM20 protein, suggesting the absence of mitochondrial clearance. Inhibition of mitophagy caused by miR-218 overexpression led to ROS accumulation, inflammation, and metabolic dysfunction, enabling an adequate environment for neurodegenerative diseases. Hence, miR-218 became a key mitophagy inhibitor by regulating *PRKN*, positioning it as a potential marker for cancer and neurodegenerative diseases (Rita et al., 2020).

Chronic neuroinflammation is a hallmark of many neurodegenerative diseases, including AD, in which β-amyloid (Aβ) extracellular plaques and τ proteins play key roles in this disease’s pathology and progression. The accumulation of Aβ plaques is closely linked to neuroinflammation. At the same time, τ proteins are associated with hyperphosphorylation, a process that disrupts normal τ function, ultimately leading to neuronal dysfunction and cell death, both AD characteristics. Numerous miRNAs have been identified as key contributors to AD pathogenesis due to their ability to regulate multiple mRNA targets through translational repression. Among these, miR-146a has emerged as a significant player with a deleterious, pro-inflammatory role. Kim et al. (2022) explored the role of miR-146a in the pathophysiology of AD. They quantified the expression of MiR-146a in the brain tissue from mixed glial and cortical neuronal cultures derived from the E18 rat embryos and confirmed AD patients’ temporal cortex, frontal cortex, and cerebellum. The results showed a significant upregulation of miR-146a expression, both intracellularly and within secreted extracellular vesicles (EVs). Functional analyses in primary rat glial cells revealed that miR-146a overexpression significantly impaired bioenergetic functions by reducing oxidative phosphorylation and glycolysis.

Additionally, miR-146a expression levels in various regions of the AD brain demonstrated significant correlations with the severity of the disease. Moreover, the total amount of voltage-dependent anion-selective channel 1 (VDAC1) was significantly decreased in the temporal cortices of AD, establishing a negative correlation between *VDAC1* and miR-146a, even though it is not its direct target. These findings suggest that miR-146a contributes to mitochondrial dysfunction and loss of integrity, exacerbating β-amyloid and τ-pathology in AD progression (Kim et al., 2022).

### 2.3 MitomiRs in cancer

Cancer is a disease distinguished by the abnormal and aberrant growth of malignant cells, and that can develop in almost any type of tissue or organ. This atypical cell behavior, if left untreated, leads to the incapacity of organs or systems to pursue their normal functions, leading to systemic organ failure with fatal consequences. Cancer is one of the most serious diseases, as it has high rates of morbidity and mortality worldwide, registering 19.3 million new cases and 10 million related deaths worldwide only in 2020 (Erturk et al., 2022).

One characteristic of cancer at the cellular level is the reprogramming of energy metabolism to avoid intrinsic programmed cell death of impaired cells. Under normal conditions, intracellular signaling of nuclear DNA and even mtDNA damage would activate p53, which at the same time would upregulate specific proapoptotic proteins from the B-cell lymphoma 2 (BCL-2) family. The X-associated protein from this family (Bax) would then bind to the mitochondria’s outer membrane to destabilize its permeability and allow the release of apoptosis-inducing factors that activate caspases that facilitate cell decay (Bienertova-Vasku et al., 2013; Lin et al., 2022). However, some tumor cells can bypass these control checkpoints and additionally tailor glucose metabolism and ROS production to satisfy their energetic needs and guarantee cancer progression. As mentioned previously, these metabolic processes are regulated by mitomiRs directly or indirectly, and outside the homeostatic scenario, their expression becomes abnormal, turning them into excellent biomarkers for cancer (Lin et al., 2022).

Sharma et al. (2021) identified mitomiR let-7a as an essential regulator of mitochondrial function in breast cancer. This miRNA, translocated from the nucleus to the mitochondria, modulated the expression of *ND4*, a critical component of Complex I in the mitochondrial respiratory chain. RNA immunoprecipitation and luciferase assays confirmed that let-7a directly interacted with *ND4* mRNA, reducing its stability and translation. This interaction led to the inhibition of Complex I activity in low-grade breast cancer MCF-7 cells, a model of non-metastatic breast cancer characterized by its reliance on oxidative phosphorylation (OXPHOS) for energy production. The decreased ATP production promoted a metabolic shift towards glycolysis, namely, the Warburg effect, associated with tumor growth and progression.

In contrast, in MDA-MB-231 cells, a highly metastatic glycolysis-dependent breast cancer model, let-7a activated Complex I, enhancing mitochondrial respiration and promoting OXPHOS. These findings highlight that let-7a differentially regulated mitochondrial metabolism in breast cancer cells depending on their metabolic context and stage. This capacity to modulate cellular bioenergetics positions let-7a as a crucial biomarker in disease progression and a promising candidate for addressing metabolic alterations in cancer (Sharma et al., 2021).

Chen et al. (2019) demonstrated how decreased expression of the mitochondrial-localized miR-5787 contributed to cisplatin resistance in tongue squamous cell carcinoma (TSCC). This team artificially downregulated miR-5787 in cisplatin-resistant TSCC cell lines Cal27 and Sc25, and they noticed that a reduced expression of miR-5787 negatively affected oxidative phosphorylation (OXPHOS), promoting a metabolic shift towards aerobic glycolysis, explained by the upregulation of hexokinase and pyruvate kinase isozyme. Moreover, miR-5787 inhibition increased ROS production in the chemoresistant cells. Furthermore, it was established that miR-5787 directly targeted mitochondrial cytochrome c oxidase subunit 3 (mt-*CO3*) but instead enhanced its translation to reverse chemoresistance. The study concluded that a low expression of miR-5787 could be used as a biomarker to predict resistance to cisplatin, offering new therapeutic perspectives for patients with TSCC (Chen et al., 2019).

In a similar line of research, the role of miR-2392 in chemoresistance and metabolic balance in TSCC was explored (Fan et al., 2019). This process is characterized by reduced OXPHOS and increased glycolysis, a metabolic shift mediated by miR-2392, a mitomiR transcribed in the nucleus, processed in the cytoplasm, and subsequently translocated to mitochondria. Clinically, overexpression of miR-2392 is associated with increased tumor survival, reduced apoptosis following cisplatin treatment, and worse patient outcomes. The study employed cisplatin-resistant Cal27 and SC9 TSCC cell lines. Transfection of miR-2392 mimic into both models revealed that the mitomiR overexpression led to a downregulation of mitochondrial complexes I, III, and IV activity, as well as elevated mitochondrial ROS levels, partially reduced OXPHOS, and diminished ATP production. It was also found that there was a significantly reduced expression of *ND2*, *ND4*, NADH dehydrogenase subunit 5 (*ND5*), cytochrome b (*CYTB*), *COX1*, and cytochrome c oxidase subunit 2 (*COX2*); mtDNA-encoded polypeptides that correspond to be critical components of the mitochondrial respiration system. Moreover, miR-2392 drove glycolytic reprogramming by upregulating *HK2* and pyruvate kinase M2 (*PKM2*), enhancing TSCC cell survival and proliferation under hypoxic environments and contributing to chemoresistance and disease progression. These findings highlight the role of miR-2392 as a promising therapeutic target to overcome conventional therapy resistance in TSCC and underscore its potential as a biomarker for the disease (Fan et al., 2019).

Dysregulation of mitomiRs contributes to mitochondrial dysfunction, a hallmark of aging, stress, and diseases such as cancer. Considering this, Kuthethur et al. (2022) investigated the role of mitochondrial genome-encoded miRNAs in breast cancer. They found that mitomiR-5 specifically targeted peroxisome proliferator-activated receptor gamma coactivator-1α (*PPARGC1A*), which is critical for mitochondrial DNA (mtDNA) transcription by promoting the expression of *TFAM*. As expected, increased expression of mitomiR-5 was accompanied by a decrease of mtDNA, impairing mitochondrial function and potentially promoting tumorigenesis. Additionally, mitomiR-5 was found to be more expressed in patients with grade 1 tumors compared to grade 3 tumors, suggesting that its expression might be associated with the initial stages of breast cancer development. This approach was also supported by the overexpression of mitomiR-5 in the MDA-MB-468 cell line rather than in MDA-MB-231. These findings suggest that mitomiR-5 plays a critical role in mitochondrial regulation, positioning it as a potential biomarker for early-stage breast cancer and as a diagnostic tool to stop cancer progression (Kuthethur et al., 2022).

Another study highlighted the central role of miR-181a-5p in metabolic reprogramming in hepatocellular carcinoma (HCC), a type of cancer characterized by reduced rates of OXPHOS and elevated glycolysis. MiR-181a-5p is encoded by the nuclear genome and later translocated to mitochondria, where it targets cytochrome b (mt-CYB) and cytochrome c oxidase II (mt-CO2d), both mitochondrial genes. The inhibition of these genes by mitomiR-181a-5p altered the subunits of the electron transport chain complexes III and IV, destabilizing the mitochondrial membrane potential. This OXPHOS disruption significantly decreased ATP production, pushing the cells towards a metabolic adaptation characterized by the upregulation of glycolytic enzymes hexokinase 2 (HK2) and glucose transporter 1 (GLUT1). The resultant increase in glucose uptake, accompanied by enhanced lactate production and lactate dehydrogenase (LDH) activity, revealed a stronger reliance of HCC cells on glycolysis for energy generation rather than for OXPHOS. This metabolic change allowed cancerous cells to persist and proliferate even under hypoxic conditions, promoting chemoresistance and metastasis, thereby facilitating tumor progression. Further, *in vivo* models inoculated with SMMC-7721-181a-5p cell line showed that mitomiR-181a-5p overexpression accelerated tumor proliferation, promoted glycolysis-driven tumor development, and facilitated early metastatic spread to distant organs, such as the lungs. This study exhibited that mitomiR-181a-5p was a potential disruptor of mitochondrial energy homeostasis and a key driver of oncogenic metabolic reprogramming, making it a solid prognostic marker for liver cancer (Zhuang et al., 2020).

### 2.4 MitomiRs in type II diabetes mellitus

Type II diabetes mellitus is a chronic disease characterized by uncontrolled episodes of hyperglycemia caused by the ineffective utilization of insulin hormone by the cells. Persistent occurrence of this state causes an excessive demand for the hormone, ultimately leading to pancreatic β-cells failure (Ahmad et al., 2022; Chama-Avilés et al., 2023). This disease has one of the most elevated prevalence rates worldwide, affecting 463 million people in 2019, and is expected to scale to 578.4 million in 2030 (Meneguin et al., 2021). One of the leading factors that contribute to the onset of diabetes is obesity, which is often accompanied by non-alcoholic fatty liver disease, retinopathy, cardiovascular disease, nephropathy, polycystic ovarian syndrome, gout, arthritis, and even Alzheimer’s and Parkinson’s diseases (Hassan et al., 2020; Rohm et al., 2022).

Diabetes is inherently classified as a metabolic disorder, given the cells’ incapacity to process carbohydrates adequately. When there is excess glucose in serum, this sugar rapidly oxidizes, resulting in increased NADH and ROS within the mitochondria of pancreatic β-cells. This cellular type is characterized by a decreased expression of antioxidant enzymes, leading to oxidative stress, inflammation, progression of T2DM, and related comorbidities (Paul et al., 2021). Intriguingly, numerous mitomiRs have been associated with an active regulation over the impaired metabolic processes that occur during this pathology (Vezza et al., 2021), broadening the pool of biomarkers that indicate the pathogenesis and course of T2DM but, more significantly, providing novel insights that may aid in improving the regulation of cellular metabolism, subsequently translating into target treatments against this chronic disorder.

It has recently been found that T2DM is closely associated with increased mitomiR-378a concentrations, a mitomiR targeting mt-*ATP6*, a component of the electron transport chain complex V (Durr et al., 2022). When mt-*ATP6* is successfully downregulated by miR-378a, it directly results in reduced ATP production and consequently in decreased mitochondrial bioenergetics, which in turn contributes to a reduction in the efficiency of OPHOS and to the accumulation of mitochondrial ROS. The study demonstrated that long non-coding RNA potassium voltage-gated channel subfamily Q member 1 overlapping transcript 1 (Kcnq1ot1) regulates miR-378a activity, acting as a sponge, therefore alleviating mt-*ATP6* repression. This sponging mechanism restored ATP synthase expression and mitochondrial function. These outcomes highlighted the potential of targeting the miR-378a/mt-ATP6 axis via Kcnq1ot1 overexpression to address comorbidity complications in T2DM, improve patient outcomes, and slow disease progression (Durr et al., 2022).

A comprehensive study investigated the role of miR-379 in the pathogenesis of diabetic kidney disease (DKD), a pathology known for impaired mitochondrial function and quality, which leads to oxidative stress (Kato et al., 2021). This miRNA resides in the cytoplasm, where it regulates protein involved in mitochondrial fission and health by binding to the transcript of mitochondrial fission protein 1 (FIS1) mRNA. Mir-379 knockout mice generated using CRISPR-Cas9 and wildtype (WT) controls were subjected to diabetes induction via streptozotocin (STZ). The miR-379 KO mice exhibited significant protection against DKD, as they did not experience weight loss associated with mitochondrial dysfunction under diabetic conditions, suggesting miR-379 deletion helps preserve renal, kidney, and muscle health. In addition, deletion of miR-379 enhanced adaptative mitophagy via *FIS1* activation, which improved the elimination of damaged mitochondria with the aim to preserve mitochondrial function, as well as prevent pro-fibrogenic pathways, emphasizing the miR-379 potential as a biomarker for DKD and offering potential avenues for therapeutic intervention (Kato et al., 2021).

### 2.5 MitomiRs in other chronic diseases

Persistent low-grade inflammation is thought to contribute to the development of many age-related diseases and is considered a central axis to develop multiple morbidities. MitomiR-181a has been shown to modulate the expression of both pro and anti-inflammatory cytokines, as well as T-cell differentiation and proliferation. Iannone et al. (2022) investigated the involvement of miR-181a in age-related chronic inflammation and the progression of multimorbidity in older adults. Expression levels of this mitomiR in plasma samples from individuals aged 65–97 years were quantified by qPCR, finding increased expression in older men and, in particular, showed a significant correlation with worsened inflammatory blood parameters in women. In addition, a positive correlation between miR-181a expression and overall multimorbidity burden was observed. These findings highlight miR-181a as a potential biomarker for assessing health status and its association with age-related chronic inflammation. However, further research was required to establish targets and regulators with the aim of finding novel insights about the signaling pathways that would explain the observed pathological associations (Iannone et al., 2022).

Major depressive disorder (MDD) is closely linked to mitochondrial dysfunction, triggering systemic inflammation. In this context, Ogata et al. (2023) identified mitochondrial-encoded microRNAs hsa-miR-6068, hsa-miR-939-5p, hsa-miR-187-5p, hsa-miR-7110-5p, and hsa-miR-4707-3p, crucially linked to mtDNA. For instance, hsa-miR-6068 regulates aldehyde dehydrogenase (*ALDH2*) and DOT1-like histone lysine methyltransferase (*DOT1L*), both involved in antioxidant responses and oxidative stress, and their suppression causes mitochondrial dysfunction. This study also stated that treatment with mirtazapine (MIR) helped reestablish hsa-miR-6068 expression and improve emotional outcomes. Overall, dysregulation of the aforementioned miRNAs was associated with chronic mitochondrial dysfunction that exacerbated MDD symptoms. These findings underscore the importance of miRNAs during the development of depression disorder and its link to mitochondrial control, highlighting them as biomarkers for the diagnosis of this disorder and their potential for developing personalized psychiatric therapies (Ogata et al., 2023).

Additionally, mitochondrial dysfunction has been increasingly recognized as a crucial contributor to the pathophysiology of asthma, a chronic respiratory disease characterized by constant airway inflammation. Recent evidence highlighted nuclear-encoded miR-128-3p as a key regulator for modulating mitochondrial dynamics that leads to the alleviation of airway inflammation. MiR-128-3p targets nuclear-encoded sine oculis homeobox homolog 1 (*SIX1*), a critical regulator of mitochondrial fission and inflammatory responses. To demonstrate this, Liu D. et al (2024) applied agomiR miR-128-3p treatment to SPF mice, which exhibited reduced inflammatory cell infiltration and collagen deposition around the airways and blood vessels, improving airway inflammation. Further molecular assays in asthmatic mice determined mitigation of SIX1 protein expression along with miR-128-3p enhanced expression. Additionally, in an *in vitro* experiment that used IL-13-stimulated bronchial epithelial cells, BEAS-2B proved that overexpression of miR-128-3p triggers SIX1 knockdown, mitigating mitochondrial division and enhancing its membrane potential, leading to lower ROS production. These findings underscored miR-128-3p as a promising therapeutic target for alleviating mitochondrial dysfunction and inflammation in asthma, offering new insights into the molecular mechanisms underlying this chronic disease (Liu X. et al., 2024).

**FIGURE 3:**
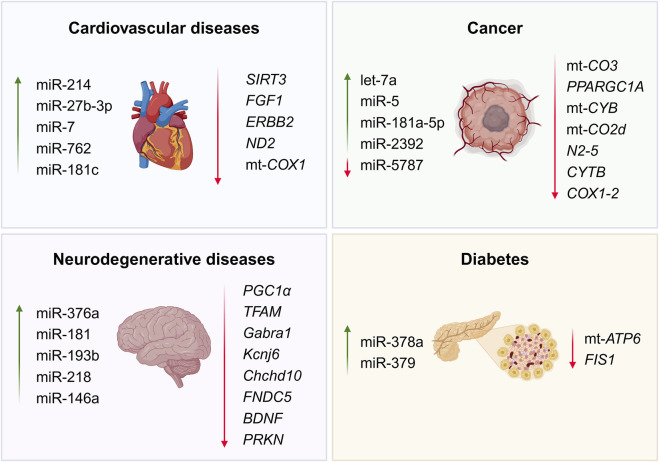
Commonly dysregulated mitomiRs and target genes in chronic diseases (Created at https://BioRender.com).

A list of mitomiRs discussed in this review is presented in Table 1, along with their target genes, function, and classification. Also, Figure 3 provides a visual insight into the presented dysregulated miRNAs and target genes within the most recurrent chronic diseases.

**TABLE 1:** Dysregulated mitomiRs in common chronic diseases.

MitomiR	Related chronic disease	Target genes	Function	References
miR-214	Cardiac hypertrophy and heart failure	*SIRT3*	MiR-214 downregulates *SIRT3*, contributing to hyperacetylation of mitochondrial proteins and disrupted energy metabolism, leading to cardiac hypertrophy and heart failure	Ding et al. (2020)
miR-27b-3p	Cardiac hypertrophy	*FGF1*	MiR-27b-3p downregulates *FGF1*, causing dysregulated mitochondrial function, myocardial fibrosis, hypertrophy, and cardiac inflammation	Li et al. (2022)
miR-7	Cardiac dilation	*ERBB2*	MiRNA-7 targets *ERBB2*, causing a disruption of mitochondria’s morphology and alterations of OXPHOS, leading to heart dilation	Gupta et al. (2021)
miR-762	Ischemia	*ND2*	MiR-762 targets *ND2*, directly affecting complex I enzyme activity, thus decreasing ATP production and increasing ROS accumulation, causing apoptotic responses	Yan et al. (2019)
miR-181c	Heart failure	mt-*COX1*	Downregulation of mt-*COX1* by miR-181c targeting causes ROS accumulation, which oxidizes cysteine residues of Sp1, thus interfering with the translation of MICU1, which causes the accumulation of calcium ions in the mitochondria	Roman et al. (2020)
miR-376a	PD	*PGC1α, TFAM*	Aberrant overexpression of miR-376a downregulates mitochondrial gene expression, inhibiting the production of respiratory chain proteins and allowing the exposition of neurotoxins	Baghi et al. (2020)
miR-181	PD	*Gabra1, Kcnj6, Chchd10*	Overexpression of miR-181 enhances neurodegeneration, toxicity vulnerability, and reduced mitochondrial function	Stein et al. (2022)
miR-193b	PD	*PGC1α, FNDC5, BDNF, TFAM*	The constant expression of miR-193b promotes ROS production, causing mitochondrial stress and neuronal degeneration	Baghi et al. (2021)
miR-218	Neurodegeneration	*PRKN*	Upregulation of miR-218 inhibits mitophagy, allowing ROS accumulation, inflammation, and metabolic impairment	Rita et al. (2020)
miR-146a	AD	-	Aberrant overexpression of miR-146a impairs OXPHOS and glycolysis, enabling mitochondrial dysfunction and contributing to neuroinflammation	Kim et al. (2022)
let-7a	Breast cancer	*ND4*	Let-7 expression blocks complex I expression, preferring the glycolysis pathway in MCF-7 cells	Sharma et al. (2021)
miR-5787	TSCC	mt*-CO3*	Downregulation of miR-5787 promotes the shift from OXPHOS to aerobic glycolysis, accumulating ROS and promoting chemoresistance	Chen et al. (2019)
miR-5	Breast cancer	*PPARGC1A*	Enhanced expression of miR-5 inhibits transcription of mtDNA by promoting the expression of *TFAM*, impairing mitochondria, and promoting initial tumorigenesis	Kuthethur et al. (2022)
miR-181a-5p	Liver cancer	mt*-CYB,* mt*-CO2d*	Overexpression of miR-181a-5p enhances the preference towards glucose uptake, inhibiting OXPHOS and facilitating tumor progression and metastasis	Zhuang et al. (2020)
miR-2392	TSCC	*ND2-5, CYTB, COX1-2*	Upregulation of miR-2392 induces a metabolic shift from OXPHOS to glycolysis, reducing ATP production and enhancing cell survival and proliferation under hypoxic environments, aiding chemoresistance	Fan et al. (2019)
miR-378a	T2DM	mt*-ATP6*	Upregulation of miR-378a reduces ATP production, causing impaired bioenergetics and ROS accumulation, promoting T2DM and associated morbidities	Durr et al. (2022)
miR-379	T2DM	*FIS1*	Upregulation of miR-379 promotes weight loss, inhibition of mitophagy processes and enhances pro-fibrogenic pathways	Kato et al. (2021)

While there is no concretely designed and straightforward screening technique for evaluating all mitomiRs that would indicate them as a biomarker by displaying their over- or under-expression, there exists a vast collection of molecular analyses that are individually designed and applied to detect prospective mitomiR biomarkers. Most of the reports here described include RNAseq, for evaluating the robustness of the expressed miRNAs; qRT-PCR, for amplifying the selected pool of mitomiRs that are of special interest; Western blotting, for detecting and quantifying the target proteins; designed microarray chips for analyzing the expression profiles of certain miRNAs; and fluorescence *in situ* hybridization, for detecting the specific localization of a desired miRNA (Roman et al., 2020; Yamada et al., 2020a; Gupta et al., 2021; Hollenberg and Singer, 2021; Li et al., 2022). These detection methods are specifically designed to be aligned with the objectives of each experiment, whether they focus on cell cultures or live models, allowing us to understand the changes in the expression profile of the mitomiRs from healthy to diseased samples. Once researchers have demonstrated through multiple preclinical techniques that a mitomiR is consistently and significantly dysregulated in a specific disease, they could take further steps to develop a precise clinical test. This test would efficiently detect abnormal mitomiR expression and ultimately aid in disease prognosis. However, multiple technical barriers must be overcome before we can reach this stage, including miRNA expression consistency, refinement of high-affinity probes, exclusivity of the marker, and protocol standardization.

## 3 Emerging mitomiR-based therapeutic strategies for chronic conditions

It is well-established that miRNAs are excellent biomarkers for a broad series of diseases, including those related to the dysfunction of the mitochondria (Sekar et al., 2020). The recent discovery of mitomiRs has drawn the attention of researchers to explore in detail their comprehensive role in metabolic pathways that have been directly linked to the progression of chronic diseases, with the aim of developing novel molecular-based treatments against these conditions, taking into advantage the capacity mitomiRs have to regulate mitochondrial-associated genes (Moawad et al., 2024). Precisely, dysregulation of mitomiRs strongly influences glycolysis (Mirzaei and Hamblin, 2020), oxidative phosphorylation (Liu et al., 2021), redox balance (Carbonell and Gomes, 2020), calcium homeostasis (Giusti et al., 2018), mitophagy (Strappazzon, 2020), and programmed cell death (Gohel et al., 2023), in a negative way, contributing to the progression of metabolic-associated chronic diseases, as all of them are key processes for a healthy metabolism. In this means, modulating the expression of mitomiRs holds promise for the engineering of novel therapeutic interventions against metabolic chronic diseases (Moawad et al., 2024).

The targeted delivery of miRNA therapeutics to specific tissues is crucial, as it allows precise modulation of mitochondrial gene expression in affected organs or cells while minimizing off-target effects, enhancing efficiency, and reducing toxicity and immune activation. (Mitchell et al., 2020). Multiple strategies have been developed to deliver miRNAs to specific cells, tissues, or organs. These systems must reunite a series of characteristics that would allow for cargo stability, high loading capacity, biocompatibility, immunoescape, and extended circulation half-life (Bravo-Vázquez, et al., 2023b; Yashooa et al., 2025). The most popular tools for nucleic acid delivery include viral and nonviral vectors. The formers have shown disadvantageous performance, as they have limited packaging, immunogenic responses, and inflammatory responses. The latter, on the other hand, includes a large variety of nanocarriers, such as liposomes, polymer-based nanostructures, and organic and inorganic nanoparticles (Liang et al., 2022). Small- and large-scale nanoparticle synthesis has garnered special attention throughout recent years, which has acceleratingly led to a broader and more comprehensive understanding of their mechanisms, applications, and therapeutic potential (Radmanesh et al., 2021; Wang et al., 2021; Kara et al., 2022; Liu et al., 2022; Yang et al., 2022). Multiple nanocarriers have been engineered for tissue-specific delivery while systemically delivered. For instance, proteins or parts of them can be attached to the nanoparticles for their recognition by tissue-specific receptors on cells’ surface; other targeting moieties include antibodies, simple sugars, glycoproteins, vitamins, transporters, and specific ligands. This selective binding interaction allows for systemic nano-treatments decorated with functional molecules to be uptaken by desired cells. Then, if required, subsequent intracellular delivery to specific compartments like the nucleus or mitochondria has been achieved with pH-responsive strategies (Mitchell et al., 2020). Although most of the current mitomiR-based treatments utilizing nanocarriers have not prioritized intracellular-specific delivery to the mitochondria, they have demonstrated effective results in mitigating chronic diseases, even if only having an approach to targeting specific organs or cells.

It has been evidenced that constant interruptions of blood supply in the kidneys (ischemia/reperfusion injury) lead to oxidative stress caused by the accumulation of reactive oxygen species (ROS), especially in renal tubules. Thereafter, this physiological imbalance results in mitochondrial fragmentation, cell death, acute kidney injury, and, ultimately, an increased incidence of chronic kidney disease (Cao et al., 2020; Ortega-Trejo et al., 2022). Various therapeutic approaches have been tested to stimulate the antioxidant capacity of mitochondria under these conditions. A recent investigation evaluated the potential of miR-200a-3p to restore the mitochondrial function in renal proximal tubular epithelial cells under induced oxidative stress. Mesenchymal stem cells’ extracellular vesicles loaded with miR-200a-3p were cocultured with cell line HK-2 treated with H_2_O_2_. Results revealed significant restoration of the mitochondria, adequate membrane potential, decreased ROS levels, augmented ATP production, and consistent mtDNA replication. Further research identified miR-200a-3p to target Kealch-like ECH-associated protein 1 (*Keap1*), a negative regulator of nuclear factor erythroid-related factor 2 (*Nrf2*). *In vitro* and *in vivo* downregulation of *Keap1* and upregulation of *Nrf2* stimulated the expression of superoxide dismutase 2 (*SOD2*), an antioxidant protein, confirming the regulatory importance of miR-200a-3p to facilitate kidney repair, potentially reducing the risk of CKD development (Cao et al., 2020).

Similarly, CKD is tightly associated with mitochondrial dysfunction in the kidneys’ proximal tubular cells. The maintenance of tubular reabsorption and secretion is a housekeeping process that allows electrolyte balance and demands high levels of energy consumption. If a mitochondrial dysfunction occurs, such as disruption of oxidative phosphorylation, it further results in decreased ATP production, destabilization of the mitochondria membrane potential, overproduction of ROS, and ultimately apoptosis. MitomiR-214 was identified as a nuclear-encoded ncRNA that translocates to the mitochondria, as it was consistently present in the mitochondrial fractions of various murine CKD-induced models. It was further noticed that this mitomiR directly targets mitochondrial genes NADH dehydrogenase 6 (mt-*Nd6*) and NADH-ubiquinone oxidoreductase chain 4L (mt-*Nd4l*), both essential for the structure of the enzymatic complex I, which plays a key role in oxidative phosphorylation. The administration of anti-miR-214 in CKD mice reduced the expression of mitomiR-214, mimicking the phenotype of wild-type models. The symptomatic consequences included the attenuation of tubular degradation, reduction of ROS production, amelioration of mitochondrial morphology, and the impediment of cellular apoptosis. This data showed the pharmacologic inhibition of mitomiR-214 through its molecular antagonist effectively reverts tubular injury in CKD (Bai et al., 2019).

The expression of a selected pool of mitomiRs was analyzed in different hepatocellular carcinoma (HCC) cell lines, consistently encountering miR-518d-5p as the most upregulated one. This mitomiR is known for targeting transcription factor c-Jun, which has p53 upregulated modulator of apoptosis (PUMA) as its mitochondrial target, a well-known pro-apoptotic gene. MiR-518d-5p has been associated with declined survival of HCC-positive patients, as well as with marked resistance to the anticancer drug sorafenib. This background led to the administration of anti-miR-518d-5p to human hepatoma BCLC3 cells, detecting increased ROS production and higher levels of c-Jun at both the transcription and translation levels. By inhibiting miR-518d-5p, the silencing of the *c-Jun* gene is released, resulting in an increase in its transcription and translation. *c-Jun*, as a transcription factor of the Activator Protein 1 (*AP-1*) oncogene, is activated in response to oxidative stress and regulates genes involved in apoptosis and cell proliferation. All of these molecular shifts caused the reduction of tumor proliferation, anti-apoptotic markers, and cancerous cell survival.

Meanwhile, the transfection of miR-518d-5p mimic to Huh7 human hepatoma cells caused the opposite effects, as the mitochondrial analyses indicated less ROS production, downregulation of c-Jun, and decreased activation of c-Jun N-terminal kinases. Finally, a complementary experiment performed on BCLC3 cells evaluated the synergistic effects of anti-miR-518d-5p and sorafenib, encountering an enhanced expression of c-Jun/PUMA and a 1.4-fold increase of apoptotic cells via induction of ROS-mitochondrial stress and ATP depletion. These findings established anti-miR-518d-5p as a therapeutic ncRNA able to treat HCC, given its capability to regulate ROS production in the mitochondria (Fernández-Tussy et al., 2021).

Inadequate management of diabetes is highly likely to result in additional comorbidities, cardiovascular complications being the most common, as they account for nearly 75% of deaths in diabetic patients. MitomiR-92a-2-5p expression is markedly decreased in cardiomyocytes during diabetic cardiomyopathy. To further determine its potential effects, its mimic was delivered to rat H9c2, murine HL-1, and neonatal rat ventricular cardiomyocytes (NRVCs) models using recombinant adeno-associated virus, finding an increase of mitochondrial gene Cytb expression in all of them, confirming that miR-92a-2-5p was able to translocate into the mitochondria to counteract Cytb downregulation. Moreover, it was observed that ROS levels decreased, enhancing mitochondrial translation, diminishing lipid deposition, and preventing apoptosis, which finally led to an amelioration of diabetic cardiomyopathy. These findings established the possibility of a new molecular-based therapeutic strategy against this cardiovascular disease (Li et al., 2019).

Idiopathic pulmonary fibrosis is a chronic disease characterized by the thickening and stiffness of the lung tissues. Recent findings have shown that during the progression of this condition, there is a marked metabolic reprogramming linked to mitochondrial autophagy in bronchi and alveoli. Molecular analyses have revealed that miR-33 levels increased in the cells of these tissues. This mitomiR has been proven to sustain cardiac and hepatic fibrosis as it promotes the proliferation of fibroblasts, preserves cholesterol, and endorses fatty acid oxidation. In this context, Ahangari and team (2023) systemically and directly administered anti-miR-33 in pulmonary macrophages using a synthetic peptide backbone as a dual protective and delivery vehicle. This treatment increased the expression of *PGC1α* and ATP binding cassette subfamily A member1 (*ABCA1*), indicating a key role of miR-33 in regulating several mitochondrial pathways. Physiological consequences included an improved oxygenation rate, enhanced autophagy, and biogenesis homeostasis. They decreased extracellular mtDNA, ultimately attenuating pulmonary fibrosis in different *in vivo* and *ex vivo* murine and human models, indicating that anti-miR-33 could regulate immune-metabolic responses of macrophages. This efficient, targeted molecular therapy is proposed to be further applied to humans with idiopathic pulmonary fibrosis (Ahangari et al., 2023).

Until now, the only successful nanocarrier reported to effectively deliver mitomiRs to the mitochondria and target this organelle’s genes is that one by Maghsoudnia et al. (2020). However, there have been other strategies for RNA delivery to this organelle, adapted from mitochondrion-targeted nanocarriers employed to carry small anticancer molecules. The most popular one consisted of a non-cationic liposome decorated with high-density octa arginine, which enters the cell via micropinocytosis, and once endosomal escape has been achieved, it binds the mitochondria electrostatically and releases its cargo (Yamada et al., 2008). So far, this nanocarrier has been used to deliver antisense RNAs, tRNAs, rRNAs, and mRNAs (Kawamura et al., 2019; Kawamura et al., 2020; Yamada et al., 2020a; Yamada et al., 2020b). However, one of its disadvantages includes a different uptake efficiency between cell lines. Particularly, the internalization of this nanodevice into cardiomyocytes has been inefficient (Yamada et al., 2020). Another strategy that has been explored to deliver RNAs to the mitochondria has been the modification of the nucleic acid sequences to make them recognizable by the mitochondria import machinery; notwithstanding, this broadens the possibility for off-targeting effects. Beyond any doubt, more extensive studies are required for the refinement of these strategies to achieve specific tissue targeting and even direct mitochondrial delivery when deemed necessary (Chernega et al., 2022).

While successful cases of RNA delivery using nanocarriers, such as the mRNA vaccines developed against COVID-19 by Pfizer-BioNTech (Polack et al., 2020) and Moderna (Baden et al., 2021) have been reported, studies that have moved into drug development programs or clinical trials for the targeted delivery of miRNAs are still scarce (Zhou et al., 2018). In fact, no miRNA-based therapeutic strategies have yet been approved by health regulatory agencies to be used as standard clinical treatments (Brillante et al., 2024). However, from both preclinical and clinical perspectives, anti-microRNA therapies have demonstrated significant potential in targeting disease-specific molecular pathways. In 2020, during phase 1b clinical trials, antimiR-132 was randomly administered to patients with reduced left ventricular ejection fraction after myocardial infarction. This therapeutic, commercially known as CDR132L, sought to inhibit the expression of miRNA-132 in cardiomyocytes, reverting maladaptive cardiac remodeling, transformation, and hypertrophy, ultimately preventing heart failure. Results showed safety and well-tolerability, parallel to miR-132 reduction in plasma in a dose-dependent manner, reduction in fibrosis markers, reduction in the amino-terminal fragment of pro-brain natriuretic peptide (NT-proBNP), which is a biomarker used to monitor heart failure, and narrowing of the QRS complex, which meant an enhancement in ventricular depolarization (Täubel et al., 2020). This phase successfully displayed clinical benefits in chronic HF patients and has moved on to phase 2 trials (Viereck and Thum, 2023). Likewise, Miravirsen, an anti-miR-122 therapy, has advanced to human trials for the treatment of Hepatitis C Virus (HCV) infection. By targeting miR-122—a microRNA essential for HCV replication—Miravirsen effectively reduced viral loads in patients, marking one of the first successful clinical applications of anti-miR therapeutics (Titze-de-Almeida et al., 2017). Another potential mitomiR-based treatment is being developed by miRagen Therapeutics; their medication, MGN-5804, is an antimiR that seeks to target and silence miR-378 for the treatment of cardiometabolic disease. So far, it has been shown that the under-expression of this miRNA ameliorates oxidative capabilities and enhances fatty-acid metabolism in the mitochondria (Chakraborty et al., 2017; Chakraborty et al., 2021). On the other hand, not all miRNA-targeting efforts have succeeded. For example, Genzyme, a Sanofi company, tested Lademirsen, an anti-miRNA-21, in patients with renal dysfunction derived from Alport syndrome (Smartpatients, 2024). This disease is well known to be affected by mitochondrial dysfunction since it contributes to podocyte deterioration and impairs lipid metabolism (Kim et al., 2024). Unfortunately, although the anti-miRNA was overall tolerated during phase I, no significant improvement in kidney function was observed, and the trial was quit at phase II (Gale et al., 2024). Despite the current efforts to advance towards the final clinical trials, significant challenges remain to be surmounted before this transition can be fully overcome.

## 4 Future insights

Despite the recent research and breakthroughs of mitomiRs since their discovery in 2009 (Kren et al., 2009), much information about them remains elusive, particularly regarding the biogenesis pathway that follows mitochondrial-originated miRNA (Luo et al., 2023), importation mechanisms from nuclear-encoded miRNAs into the mitochondrial compartments (Erturk et al., 2022), possible gene regulation effects, mechanisms of action, and phenotypic consequences. MtDNA-encoded mitomiRs that have been primarily predicted using mitochondrial transcriptome analyses and bioinformatic tools, yet much experimental work must be done in order to confirm their synthesis mechanism and functional processing for complete maturation (Luo et al., 2023). The current available empirical studies that have studied the presence of miRNAs encoded by the mitochondrial genome have focused their strategies on depleting all mitochondrial DNA that downregulated all mtDNA-encoded transcripts. Their results suggested that mitochondrial transcription machinery is crucial for mitomiR expression and probably that mitomiRs are encoded by the mitochondrial genome (Kuthethur et al., 2022). Other authors have suggested premiR-let7, pre-miR-302a, miRNA-1974, miRNA-1977, and miRNA-1978 as directly transcribed from the mitochondrial DNA, as they have exhibited perfect complementarity with mitochondrial genes; nonetheless, all of them mentioned that their findings merely validate the co-localization of these miRNAs inside the mitochondria, but they could not elucidate if they were endogenously synthesized, encouraging further studies upon this topic (Canale and Borghini, 2024).

Another challenge when exploring mitochondrial-encoded miRNAs is the absence of miRNA processing enzymes, which participate in the miRNA biogenesis canonical pathway within the mitochondrial compartments. The lack of evidence that backs up the presence of Drosha and Dicer proteins in the mitochondria has implied alternative non-canonical biogenesis pathways (Canale and Borghini, 2024), including Drosha-independence, which has been confirmed for mirtrons, byproducts of intron cleavage during the alternative splicing process (Rorbach et al., 2018). In this way, it is crucial to provide compelling experimental evidence demonstrating miRNA biogenesis originating from mitochondrial DNA. Further investigation in this area is deemed valuable, as it would become a paramount validation for ncRNA and mitochondrial science.

It is worth mentioning that, besides miRNAs, other regulatory ncRNAs have been discovered to influence mitochondrial processes and, more importantly, have regulatory functions inside this organelle. The first long non-coding RNA found in the mitochondria was discovered in 2007 (Villegas et al., 2007), where they determined that its synthesis required mitochondrial transcription and that it was co-localized with the mitochondrial exclusive proteins cytochrome c and endonuclease G of proliferating cells. Furthermore, this 2374 nucleotide transcript revealed the presence of an inverted repeat of 815 base-pairs linked to the 5′end of the mitochondrial 16S ribosomal RNA- Together, they assemble in a double-stranded DNA, conferring resistance against RNases. Four years later, lncCytb, lncND5, and lncND6 were found to be particularly enriched in the mitochondria (Rackham et al., 2011), generated from the mitochondrial genome, processed by nuclear-encoded proteins and mitochondrial enzyme RNase P protein 1, and more certainly, have a role in regulating mitochondrial gene expression (Ren et al., 2023). Moreover, various circular RNAs (circRNAs) have also been found to interact with mitochondria stability, whether they were nuclear encoded or originating from mtDNA. For instance, CircPUM1, generated from the Pumilio RNA Binding Family Member 1 (*PUM1*) gene on human chromosome 1, was localized in the mitochondria of esophageal squamous cell carcinoma (ESCC) cell lines and stood out as a mediator of Ubiquinol-cytochrome c reductase core protein I and II (UQCRC1/2), core proteins of complex III; thus, playing a key role in ATP production. On the other hand, mitochondrially encoded circRNAs mecciND1 and mecciND5, derived from *ND1* and *ND5*, respectively, were confirmed as protein trafficking mediators via interaction with TOM40. Moreover, in hepatocellular carcinoma, both mecciRNAs had an evident upregulated expression (Liu X. et al., 2024).

Although the discovery of the tight interaction between miRNAs and multiple mitochondrial functions is relatively new, therapeutic alternatives focused on the specific delivery of mitomiRs into the mitochondria have already been developed. In 2020, a mimic of mitomiR let-7b was loaded into PANAM dendrimers decorated with triphenylphosphonium cation (TPP) and delivered into lung cancer A549 cells. The nanoparticle system was efficiently accumulated inside the mitochondrial environment, significantly reducing the expression of *MT-CO1* and *MT-CO2* genes, coding subunits of complex IV. The mitochondria-targeted nanotreatment impaired OXPHOS, increased ROS level, and inhibited non-small cell lung cancer proliferation (Maghsoudnia et al., 2020). The success of this mitochondrial-specific delivery method opens the door for the development and improvement of nano-vehicles that direct new treatments specifically to this organelle, which is involved in many drastic chronic diseases, laying the foundation for a new medical approach that focuses on mitochondrial-targeted molecular systems.

A deeper understanding of miRNAs’ impact on mitochondrial function through experimental trials will allow us to generate mitochondrial-targeted vehicles that carry more specific treatments against mitochondria-related diseases. Although there are extensive research studies around mitochondrial agomiRs and antagomiRs and their impact on chronic diseases, positioning them as notable biomarkers, their precise application as treatments is immensely underexplored. To the best of our knowledge, except for that study performed by Maghsoudnia et al. (Maghsoudnia et al., 2020), there are no other research efforts that focus on the specific delivery of miRNAs to the mitochondria to regulate the expression of transcripts encoded by the mtDNA.

It is worth mentioning that the consistency of mitomiR expression patterns can vary significantly across different patient populations, age groups, and ethnic backgrounds. Several studies have begun to address these differences, providing valuable evidence on how these factors influence mitochondrial biology. A study conducted by researchers at the Roswell Park Comprehensive Cancer Center examined microRNA expression in breast cancer, comparing women of African and European ancestry. Using sequencing techniques, they identified 102 differentially expressed miRNAs related to estrogen receptor status, with only 23 being shared between both groups. This finding highlights unique and specific expression patterns for each ethnic group, suggesting that ancestry may play a key role in the biological profile of miRNAs (Gong et al., 2019). In another study, miRNA expression was investigated in breast cancer patients across different age groups, with a particular focus on Asian women. The study found that the age at diagnosis influenced miRNA expression profiles, with significant variations observed among very young women (<35 years), young women (36–40 years), premenopausal women (41–50 years), and postmenopausal women (>50 years) in both tumor and normal tissue samples. These findings suggest that age not only impacts miRNA expression patterns but also affects clinical characteristics and patient prognoses (Tsai et al., 2018). These marked differences in mitomiR expression across diverse populations highlight the need for further research to avoid generalized assumptions of mitomiR expression in a certain pathology and, in this way, ensure more accurate disease modeling. Future studies should focus on elucidating population-specific mitomiR profiles to refine diagnostic and therapeutic strategies, ultimately advancing the field toward a more precise and personalized medicine approach.

Regardless of these differences, there is mounting evidence that mitomiRs, being nuclear or mt-DNA encoded, translocated, or acting upon cytoplasmatic mRNA, holds an essential role in controlling diverse mitochondrial activities indispensable for maintaining the cell’s integral and controlled function. Accordingly, orchestrating their expression to fit situational requirements corresponds to paramount therapeutic opportunities against the progression of chronic diseases.

## 5 Concluding remarks

Mitochondria are core regulators of many of the most important processes of the cell, including ATP production, ROS scavenging, redox balance, and apoptotic signaling processes, turning them into target organelles to treat metabolic-related diseases. As a matter of fact, multiple chronic diseases have been associated with mitochondrial dysregulation but more importantly, with the impairment of miRNAs that control the mitochondrial dynamics. This interaction has positioned them as authentic biomarkers of chronic pathologies such as neurodegenerative and cardiovascular diseases, diabetes, asthma, cancer, and even depression. Moreover, the capacity of mitomiRs to interfere with the expression of many genes involved in mitochondrial processes has designated them as specific therapeutic targets that would alleviate the progression of chronic health issues linked to cellular metabolism.
